# Beyond Reanalysis: Critical Issues in Data Reuse for Solid Tumor Proteomics

**DOI:** 10.3390/proteomes14020016

**Published:** 2026-04-07

**Authors:** Federica Franzetti, Nicole Giugni, Manuel Airoldi, Heather Bondi, Tiziana Alberio, Mauro Fasano

**Affiliations:** 1 Department of Science and High Technology, University of Insubria, 21052 Busto Arsizio, Italy; ffranzetti@uninsubria.it (F.F.); ngiugni@uninsubria.it (N.G.); mairoldi@uninsubria.it (M.A.); heather.bondi@uninsubria.it (H.B.); mauro.fasano@uninsubria.it (M.F.); 2 Department of Medicine and Technological Innovation, University of Insubria, 21100 Varese, Italy; 3 Neuroscience Research Center, University of Insubria, 21052 Busto Arsizio, Italy

**Keywords:** proteomics data reuse, data harmonization, solid tumors, data standards, precision oncology, public repositories, proteoforms

## Abstract

Proteomics represents a fundamental layer for understanding the molecular complexity of solid tumors by quantifying protein abundance and capturing proteoforms and post-translational modifications undetected in genomics or transcriptomics analyses. As mass spectrometry-based technologies and public proteomics repositories have expanded, opportunities for large-scale data reuse have grown accordingly. Nevertheless, data availability has not been translated into straightforward reuse: differences in experimental design, acquisition strategies, quantification workflows and metadata quality still limit the reproducibility and cross-study comparability. In this review, proteomics data reuse is defined as the systematic reanalysis and integration of publicly available datasets to support precision oncology applications such as biomarker assessment and antibody–drug conjugate target prioritization. We discuss reuse as an end-to-end analytical process, focusing on data analysis workflows, harmonization strategies, and the impact of heterogeneous experimental and analytical choices on interoperability. The increased application of artificial intelligence in proteomics data integration and reuse is also addressed, highlighting its analytical potential while underscoring the risks of overinterpretation when biological context and data structure are not adequately considered. Using colorectal and prostate cancer as representative examples, we illustrate how proteomics data reuse can support biological discovery and translational research, while critically examining the factors that limit robustness and clinical relevance.

## 1. Introduction

Proteomics plays a crucial role in cancer research by providing insights into protein abundance, post-translational modifications (PTMs), and molecular interactions that are often overlooked by genomic or transcriptomic analyses. Mass spectrometry (MS)-based technologies, widely regarded as the gold standard in proteomics, enable high-throughput proteome profiling and are extensively used in discovery-driven cancer studies [1,2]. In contrast, affinity-based techniques rely on highly specific molecular interactions and offer increased precision for targeted protein measurements, making them particularly suitable for validation studies and focused analyses in cancer biology [2,3,4].

Alongside these technological advances, the reuse of public proteomics datasets has gained increasing attention as a powerful strategy to extend the impact of existing data in cancer research. Proteomics data reuse can be broadly defined as the systematic reanalysis and integration of publicly available datasets to generate new biological or clinical insights beyond the original scope of individual studies. Through cross-cohort validation, meta-analyses, and large-scale integrative approaches, data reuse has the potential to improve biomarker discovery and support therapeutic target identification thereby enhancing translational relevance [5,6,7].

Major public repositories provide access to a rapidly growing number of high-quality, tissue-specific datasets, thus providing a valuable foundation for large-scale reuse initiatives [8,9,10,11]. However, despite their considerable potential, the effective reuse of proteomics data remains challenging. Differences in experimental design, acquisition strategies, data processing pipelines, and annotation practices can substantially limit reproducibility and cross-study comparability if not explicitly addressed.

**Figure 1:**
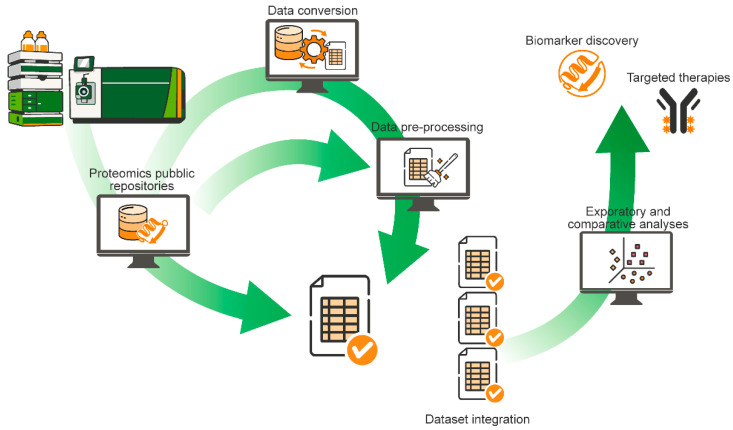
Overview of the workflow for proteomics data reuse. Public proteomics datasets are accessed from dedicated repositories and are available at different levels of data formats. These range from raw data requiring conversion and pre-processing to partially processed data requiring only pre-processing, and fully pre-processed data ready for downstream analyses. Following quality assessment, datasets from multiple independent studies may be integrated and subjected to statistical and exploratory analyses. Through cross-cohort analyses, proteomics data reuse supports the validation of protein-level signals, strengthens biomarker discovery, guides targeted antibody-based therapies development and contributes to a deeper understanding into tumor biology.

In this review, proteomics data reuse is considered not as a single analytical step, but as an end-to-end workflow (Figure 1). Within this framework, data harmonization refers to the practical effort required to make datasets generated under different experimental and analytical conditions comparable and suitable for joint analysis. Harmonization does not imply forcing datasets into uniformity, but rather acting on how data are represented, processed and interpreted. This includes alignment of data formats and metadata, management of batch effects and missing values, and the adoption of consistent quantification, normalization and filtering strategies. These steps are essential to reduce technical variability while preserving biologically meaningful signals, a balance that becomes particularly critical when reuse aims to resolve PTM- or proteoform-level information.

As a proof-of-concept, reanalysis of publicly available proteomics datasets from colorectal cancer (CRC) and prostate cancer (PC) highlights the practical difficulties associated with identifying robust and clinically actionable biomarkers for targeted therapies, including antibody–drug conjugates (ADCs). In both cases, inadequate sample filtering, variable data quality, and differences in quantification depth and coverage can lead to overgeneralized conclusions that fail to capture tumor-specific biology. Such limitations are particularly critical to ADC development, where accurate assessment of target protein abundance and specificity, proteoform diversity and tumor heterogeneity are essential for translational success.

This review aims to critically assess the current landscape of proteomics data reuse in cancer research, moving beyond simple reanalysis toward a more integrative and critical interpretative framework. It explores the major challenges associated with data integration and interpretation, spanning biological tumor complexity, technical variability, data accessibility and standardization. Particular attention is given to emerging efforts in data harmonization, standard formats and proteoform-aware analysis strategies that seek to improve the biological resolution and reproducibility of reuse studies.

Finally, the growing application of machine learning (ML) and artificial intelligence (AI) approaches in proteomics data reuse offers promising opportunities to uncover complex patterns linked to clinical outcomes [12,13,14]. While these methods hold potential for improving faster diagnosis, risk stratification, and personalized treatment strategies, their successful application critically depends on data quality, appropriate model design, and careful biological interpretation [15,16,17]. Without these prerequisites, ML-driven reuse risks reinforcing technical bias rather than revealing clinically meaningful biology.

## 2. Why Proteomics Data Reuse Is Particularly Challenging in Solid Tumors

The intrinsic biological properties of solid tumors make proteomics data highly context-dependent, posing substantial challenges for data reuse, meta-analyses, and cross-cohort integration. Unlike more homogeneous biological systems, solid tumors are dynamic and heterogeneous entities in which assumptions of molecular comparability across samples, studies, or cohorts are frequently violated.

At the genetic and epigenetic levels, solid tumors are shaped by somatic mutations, chromosomal rearrangements, copy number variations, and cell cycle deregulation, all of which directly influence protein abundance and PTMs, thereby expanding the detectable proteoform space [18,19,20,21]. Alternative splicing further increases proteomic complexity by generating multiple isoforms from a single gene, which are difficult to distinguish reliably using most conventional proteomics workflows [21]. In parallel, metabolic reprogramming, such as the preferential reliance on aerobic glycolysis, introduces additional functional variability by modulating enzyme abundance, activity, and PTM states [22]. Together, these processes contribute to substantial inter- and intratumoral variability at the protein level, complicating the reuse of datasets generated under different biological contexts.

Beyond genetic diversity, solid tumors exhibit pronounced intratumoral heterogeneity. Clonally distinct malignant subpopulations often coexist within the same tumor mass, each characterized by divergent proteomic profiles. This variability is further amplified by spatial organization: regions differing in oxygenation, nutrient availability, vascularization, or proximity to necrotic areas can display markedly distinct protein patterns and PTM landscapes [18]. As a consequence, proteomics data derived from bulk tumor samples often represent a spatially biased snapshot rather than a comprehensive molecular portrait, thereby limiting reproducibility and cross-study comparability. These limitations are particularly relevant for PTM-level analyses, where localized signaling events may not be reflected in average protein abundance measurements.

The tumor microenvironment further contributes to proteomic complexity. Solid tumors are characterized by heterogeneous cellular populations, including cancer-associated fibroblasts, endothelial cells, immune infiltrates, and extracellular matrix components, all contributing to overlapping and context-dependent proteomic signals [22,23]. Dynamic interactions between tumor cells and stromal compartments, together with gradients of oxygen, pH, and secreted factors, modulate protein abundance, PTMs, and downstream signaling pathways [19,22,23]. These effects complicate the separation of tumor-intrinsic signals from microenvironment-derived contributions in data reuse.

Immune-related variability represents another important source of proteomic divergence. Tumor immune evasion strategies, along with heterogeneous immune cell infiltration across patients, tumor regions, and disease stages, profoundly reshape tumor proteomes [24]. Moreover, tumor progression and metastatic dissemination introduce further variance: primary tumors and metastatic lesions, as well as early- and late-stage cancers, frequently display distinct proteomic signatures [19,22,23]. As a result, proteomics datasets generated at different anatomical sites or clinical stages are frequently difficult to integrate directly, even when nominally derived from the same tumor type.

Emerging single-cell and spatial proteomics approaches provide new opportunities for disentangling cellular and spatial heterogeneity, but their limited depth, sensitivity, and throughput currently constrain routine application and large-scale data reuse [25]. Efforts to generalize proteomic findings across tumors must therefore be carefully balanced against the principles of precision medicine. Excessive aggregation of datasets may obscure patient-specific molecular features that are essential for individualized therapeutic strategies. This is particularly critical for proteoform-level information. While phosphorylation-dependent signaling has been extensively characterized, other PTM classes, including glycosylation, proteolytic processing, and lipid modifications are less consistently captured in large-scale tumor datasets [26]. These imbalances reflect both technical constraints and historical research focus.

Careful consideration of these sources of heterogeneity is therefore required to support clinically meaningful, personalized applications in solid tumor research.

## 3. Proteomics Techniques: MS-Based and Affinity-Based Approaches

While the biological complexity of tumor samples presents intrinsic challenges, the technologies used to generate proteomics data introduce additional sources of variability and potential bias. Technical artifacts can arise at every stage of the experimental pipeline, potentially affecting reproducibility, comparability, and long-term reuse of proteomics datasets. The key characteristics and limitations of each technique are summarized in Table 1.

### 3.1. MS-Based Proteomics

MS-based proteomics remains the gold standard for protein identification and quantification and is the primary technology for large-scale discovery studies due to its sensitivity, dynamic range, and ability to detect PTMs [27]. MS-based approaches can be broadly categorized into top–down and bottom–up strategies.

Top–down proteomics enables direct analysis of intact proteins, revealing proteoforms resulting from genetic variations, alternative splicing, and PTMs [27,28]. However, it faces challenges like lower ionization efficiency and sensitivity for large proteins, limiting its use to specific studies or enriched samples [29].

Within this framework, integrative workflows coupling two-dimensional gel electrophoresis (2DE) with LC-MS/MS represent a high-resolution variant of top–down proteomics, in which intact proteoforms are first separated according to isoelectric point and molecular weight, prior to in-gel digestion and mass spectrometric identification, thereby enhancing proteoform resolution and reducing co-elution artifacts [30]. Advanced techniques, such as high-resolution LC-MS/MS and Gel-Eluted Liquid Fraction Entrapment Electrophoresis Mass Spectrometry (GELFrEE-MS), improve proteoform detection, although technically challenging and generally limited to smaller or enriched proteins [30,31,32,33].

Bottom–up proteomics, on the other hand, provides high sensitivity and proteome-wide coverage, but disrupts direct peptide-to-protein relationships [27,28,29] and leads to loss of proteoform-level information, making it less effective for detecting PTMs, splice variants, or sequence variants [30].

#### 3.1.1. Data Acquisition Strategies

In bottom–up proteomics, the data acquisition strategy strongly influences peptide selection, proteome coverage, and quantification reliability, thereby affecting reproducibility, data completeness, and the potential for cross-study integration and reuse [27].

The two main acquisition strategies are data-dependent acquisition (DDA), typically used for discovery-oriented experiments, and data-independent acquisition (DIA), which is particularly suited for quantitative analyses across large sample cohorts.

DDA relies on stochastic, intensity-driven precursor selection, resulting in non-random missing values, inconsistent detection of low-abundance peptides, and limited run-to-run reproducibility [27,28,29,30]. These limitations reduce dataset comparability, complicate longitudinal analyses and meta-analyses, and reduce the long-term reusability of DDA-derived data.

DIA improves quantitative consistency by systematically fragmenting all ions within predefined m/z windows, generating datasets with higher reproducibility and less missing values, which are generally more suitable for reuse [27]. However, DIA-based reuse is often constrained by computational complexity and dependence on spectral libraries, which may be incomplete, platform-specific, or heterogeneous across studies, thereby hindering harmonization.

Sequential Window Acquisition of all Theoretical Mass Spectra (SWATH-MS) enables broad proteome coverage and retrospective analysis of fragment ion maps, supporting PTM-focused studies. Nevertheless, poor standardization of acquisition protocols and instrument settings across laboratories continues to limit reproducibility and cross-study comparability.

Targeted acquisition strategies, including Selected and Multiple Reaction Monitoring (SRM/MRM) and Parallel Reaction Monitoring (PRM), provide highly reproducible and precise quantitative measurements [29]. These approaches are widely used for biomarker validation, but are inherently limited to predefined protein panels, restricting their applicability for discovery-driven applications and proteome-wide data reuse.

#### 3.1.2. Sources of Bias and Variability in Quantification Methods

Relative quantification enables comparisons of protein abundance across conditions without target selection, making it cost effective and suitable for complex systems [29,34]. Its scalability and flexibility make relative quantification particularly amenable to data reuse, as the integration of multiple independent datasets can substantially increase statistical power, improve effect size estimation, and enhance the detection of subtle but biologically relevant protein abundance changes. However, both experimental and computational challenges affect reproducibility and comparability.

Label-free quantification (LFQ) provides greater flexibility and scalability, making it conceptually well suited for data reuse [35,36]. However, LFQ is highly sensitive to variability in pre-analytical and analytical factors, which can substantially limit reproducibility and cross-study comparability if not explicitly addressed.

Label-based methods (e.g., isotope-coded affinity tags (ICAT), isobaric tags for relative and absolute quantification (iTRAQ), tandem mass tags (TMT), and Stable Isotope Labeling by Amino acids in Cell culture (SILAC)) improve within-experiment precision but introduce systematic biases, including incomplete labeling, isotopic impurities, ratio compression, that limit cross-study comparability, constraining its routine application for large-scale data reuse [29,34,37].

Absolute quantification provides biologically interpretable measurements but involves important trade-offs. Label-based approaches using synthetic peptides or proteins (e.g., absolute quantification (AQUA), protein standard absolute quantification (PSAQ), and quantification concatemers (QconCAT)) offer high specificity and accuracy but require predefined targets, extensive assay optimization, and careful control of digestion efficiency and peptide recovery [29]. Label-free absolute quantification approaches reduce cost and increase throughput but generally exhibit lower precision and greater susceptibility to technical variability and missing values [34,35], limiting robustness across studies.

Understanding these limitations is essential for designing experiments and generating datasets suitable for reliable reuse and integrative analyses.

### 3.2. Affinity-Based Proteomics Techniques

MS-based proteomics enables unbiased, hypothesis-free discovery and proteoform characterization, but its sensitivity for very low-abundance proteins in complex matrices is limited without extensive fractionation. Affinity-based platforms were developed to provide high sensitivity and throughput, particularly for targeted protein quantification. However, these technologies are constrained by target pre-selection, reagent dependency, and platform-specific biases [38,39].

Antibody microarrays and reverse-phase protein arrays (RPPAs) are limited by cross-reactivity, narrow dynamic range, and strong dependence on antibody quality and validation [39,40], resulting in variable reproducibility. Ultrasensitive platforms such as Proximity Extension Assay (PEA, Olink) and electrochemiluminescence improve detection of low-abundant proteins but are restricted to predefined panels [39,41,42], limiting discovery potential and reuse. Bead-based assays (e.g., Luminex, Simoa, and SOMAscan) offer high multiplexing and sensitivity but are affected by analyte-specific variability, matrix effects, biological heterogeneity, high costs, and limited cross-platform harmonization [39,42,43,44,45], complicating reproducibility and data integration.

Targeted proteomics bridges discovery and validation by enabling precise quantification of selected candidates. Current targeted methods cannot keep pace with the large number of hits from discovery studies, resulting in only a small fraction of candidates being validated due to time and cost constraints. Additionally, not all proteins have available antibodies, the quality of available antibodies varies significantly, and PTMs at or near the epitope can interfere with antibody binding. To address this, two antibodies may be used to target both the protein sequence and the PTM [30].

MS-based and affinity-based approaches offer complementary strengths: MS excels at unbiased discovery and molecular characterization, while PEA and SOMAscan provide sensitive, targeted quantification of low-abundance proteins. However, differences in measurement principles, output formats, and analytical biases pose major challenges for cross-platform integration and large-scale data reuse.

## 4. The Nature and Structure of Proteomics Data

**Figure 2:**
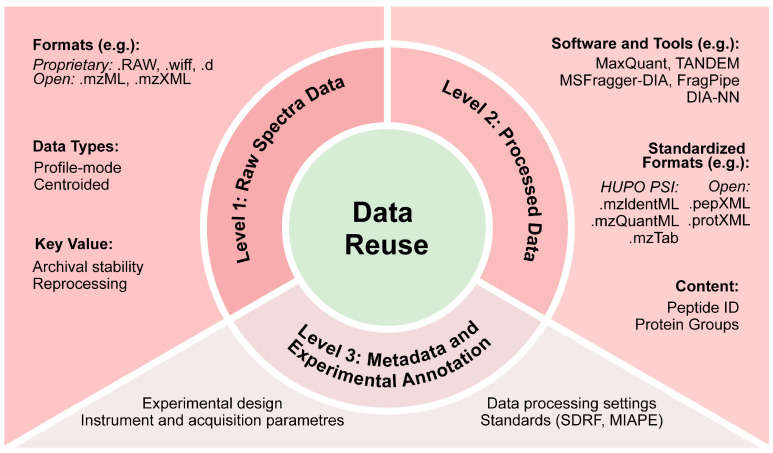
Proteomics data levels supporting reuse and integration. Proteomics data are organized into three key levels: Level 1 (Raw Spectral Data) preserves the original mass spectrometry signals; Level 2 (Processed Data) contains peptide and protein identifications and quantifications; Level 3 (Metadata) describes experimental design, instrument settings, and sample annotations. Together, these levels enable cross-study integration and large-scale reuse of proteomics data. MIAPE, Minimal Information About a Proteomics Experiment; SDRF, Sample and Data Relationship Format. File formats and software names shown are illustrative examples and are not exhaustive.

MS-derived proteomics data are typically organized into three levels: raw spectral and instrument-generated peak files (Level 1), quantified peptide- or protein-level data derived from computational processing (Level 2) and metadata describing experimental design, sample characteristics and analytical parameters (Level 3) (Figure 2) [10]. These datasets may be stored in vendor-specific or open formats; adoption of standardized formats and structured metadata, as promoted by the Human Proteome Organization Proteomics Standards Initiative (HUPO PSI), is essential for interoperability and long-term reuse [46].

### Data Types and the Evolution of Open Formats

Raw spectral data (Level 1) constitute the primary output of MS runs and are essential for data reuse. Vendor-specific formats, like .RAW, .wiff, or .d, include all instrument metadata and spectra but require proprietary software, creating potential future inaccessibility. Open and standardized formats, like .mzML, .mzXML and binary HDF5-based .mzMLb, provide software-independent access and improve long-term reusability [46,47]. Furthermore, profile-mode data keep full peak shapes for high-quality reprocessing and signal deconvolution [46,48,49], unlike centroided data that lose details [50,51]. This is relevant in proteogenomic studies, where MS data are combined with genomic and transcriptomic information, enabling the identification of tumor-specific peptides and PTMs [52,53,54]. Formats like Sample and Data Relationship Format (SDRF) ensure accurate mapping of samples to raw files and improve reproducibility across studies [55].

Processed data (Level 2) include peptide and protein identifications and quantifications derived from raw spectra through computational workflows and tools such as MaxQuant [49] and TANDEM [56]. Recent DIA-focused tools, including MSFragger-DIA, FragPipe [57] and DIA-NN [58], generate reusable datasets without access to raw files. Standardized open formats, such as pepXML and protXML, or HUPO PSI formats like mzIdentML [59], mzQuantML [60] and mzTab [61], facilitate cross-study integration. Processed datasets also enable exploration of proteoform diversity, allowing cell-type-specific protein profiling [62] and detection of proteins with altered abundance across conditions [6,55]. When coupled with modern analytical approaches, these standardized processed data facilitate the investigation of proteoforms and complex protein networks [48]. ProForma 2.0, a format developed by PSI, provides a machine-readable description of proteoforms, including PTMs, sequence variants and terminal truncations, further supporting interoperability [52,54,63].

Metadata and experimental annotations (Level 3) provide essential context for interpreting and reusing both raw and processed data [46]. These metadata include detailed information about biological samples, experimental design, sample preparation protocols, instrument settings and PTMs [46]. Community standards such as HUPO PSI and Minimal Information About a Proteomics Experiment (MIAPE) define structural frameworks for metadata representation. These standards are operationalized in formats like MAGE-TAB Proteomics, comprising Identification Definition Format (IDF) files for dataset-level metadata and SDRF-Proteomics files linking raw data to biological samples [59,64]. Properly annotated metadata enables comprehensive exploration of proteome complexity and proteoform diversity, supporting cancer proteogenomics studies [46,64,65].

## 5. Data Repositories for Proteomics Data Sharing and Reuse

The growing number of proteomics datasets remain largely inaccessible if stored locally, limiting reproducibility and secondary analyses. Deposition in public repositories is now required or strongly encouraged by funding agencies and high-impact journals, facilitating re-analysis and integrative studies [66]. Public proteomics data resources include general-purpose repositories, domain-specific platforms, and specialized repositories for proteoform-level data.

The ProteomeXchange (PX) consortium coordinates dataset submission, dissemination and indexing across multiple partner repositories [66,67]. PX promotes the adoption of community-accepted open formats (e.g., mzML, mzIdentML, mzTab), structured metadata (e.g., SDRF-Proteomics), compliance with MIAPE guidelines, and controlled vocabularies [11,60,61,68,69]. These elements support FAIR-oriented data deposition, making datasets Findable, Accessible, Interoperable, and Reusable (FAIR), and facilitating long-term reusability. By January 2026, PX had coordinated over 64,000 submissions, of which approximately 69% are public and 31% private, reflecting both accelerated technological development and increased data sharing [68,70]. ProteomeCentral serves as the central discovery portal, allowing dataset retrieval via PXD accession numbers and Universal Spectrum Identifiers (USIs) [71], while high-volume data transfer is supported through Aspera Connect and FTP [72]. All PX datasets are indexed in OmicsDI, facilitating integrative reuse across domains [68,73].

### 5.1. ProteomeXchange Repositories

PX-affiliated repositories provide standardized submission workflows and long-term data accessibility (Table 2). Proteomics identifications (PRIDE) Archive, hosted at EMBL-EBI, US-based Mass Spectrometry Interactive Virtual Environment (MassIVE), jPOSTrepo (Japan ProteOme STandard Repository), iProX (developed within the Chinese Human Proteome Project), Panorama Public and PeptideAtlas contain data from a wide range of MS approaches, like DDA, DIA, top–down, peptidomics, and crosslinking experiments [9,11,67,68,74,75,76,77,78,79,80,81,82]. All these repositories support FAIR data reuse through structured metadata, integration with protein databases and tools for data reanalysis. All these factors in PRIDE enable data reanalysis and the verification of peptides, PTMs and Single Aminoacid Variants (SAAVs) [71,75,76]. MassIVE and MassIVE-KB (MassIVE Knowledge Base) provide structured storage and traceable spectral libraries [68,78]. jPOSTrepo and jPOSTdb ensure reproducible reanalysis through standardized metadata and workflows [77,79,80]. iProX facilitates large-scale reuse and provenance tracking [77,82]. Panorama Public enables traceable submission and exploration of quantitative data [81]. PeptideAtlas organizes reanalyzed datasets into species- and project-specific builds, supporting reproducible peptide- and protein-level evidence [9]. Together, PX partner repositories provide a robust infrastructure for standardized proteomics data deposition, although effective reuse depends on repository standards, metadata completeness, data quality, and interoperable workflows.

### 5.2. Complementary Proteomics Resources

Beyond PX partner repositories, several large-scale proteomics resources provide curated and integrated datasets optimized for specific reuse scenarios. The Clinical Proteomic Tumor Analysis Consortium (CPTAC), established by the US National Cancer Institute, provides uniformly processed large-scale MS-based proteomics datasets integrated with matched genomic, transcriptomic, and clinical data from The Cancer Genome Atlas Program (TCGA) and related cohorts. Standardized analysis pipelines and structured metadata enable reproducibility, cross-study comparisons, and integrative multi-omics analyses in cancer research [8].

The Human Protein Atlas (HPA) enables reuse of spatially resolved protein level data across human tissues, cell types and subcellular compartments. By providing protein profiles, uniform experimental pipelines, explicit antibody validation, and confidence scoring, HPA supports biomarker discovery and cross-study comparisons [83].

The Pan-Cancer Proteome Atlas (TPCPA), developed by a multi-institutional international consortium, provides DIA-MS-based quantification of 9670 proteins across 999 primary cancer samples representing 22 cancer types [84]. Structured metadata and processed data allow immediate reuse without reprocessing raw files. A unified pan-cancer dataset generated by integrating multiple independent tumor cohorts enables robust cross-study comparisons and identification of cancer type-specific protein patterns [85]. In other studies, combining proteomic and transcriptomic profiles has revealed altered pathways and potential prognostic or diagnostic biomarkers [86]. Data-driven proteogenomic approaches provide processed datasets suitable for downstream analyses, including network modeling and multi-omics integration [87,88].

### 5.3. Proteoforms Data Repositories

Current efforts to enable data reuse at the proteoform level are supported by specialized repositories. The Human Proteoform Atlas (HPfA) systematically collects, curates, and disseminates human proteoforms identified primarily through top–down proteomics experiments [89]. The HPfA adopts FAIR data principles and provides structured access to proteoform-level information, including sequence variants and PTMs, thereby supporting comparison and reuse of proteoform identifications across independent studies [89]. Currently, this repository includes approximately 60,000 proteoforms across 48 dataset entries, but cancer-related studies remain underrepresented, with only two datasets derived from human tumor tissues currently included.

The Human Proteoform Project (HPP), coordinated by the Consortium for Top-Down Proteomics (CTDP), maps the human proteome at the individual proteoform level. By treating each proteoform as a distinct entity, HPP reveals molecular diversity that is often masked by traditional peptide-based aggregation [53]. These resources complement standard PX repositories, providing the necessary infrastructure for high-resolution, proteoform-aware analyses in both basic and translational research.

## 6. Downstream Challenges

Proteomics data reuse remains constrained by gaps in infrastructure, standardization, and interoperability. Many datasets fail to fully comply with FAIR principles due to incomplete metadata, inconsistent submission practices, and fragmented standards. In solid tumor studies, missing clinical annotations, unclear sample-to-raw-data mapping, and partial adoption of MIAPE guidelines [64] limit cross-study integration, reduce statistical power, and hinder reproducibility and translational applications. Without mandatory enforcement of standardized metadata frameworks, repositories may host datasets of limited value for large-scale reanalysis and precision oncology.

Data standardization and interoperability remain challenging despite HUPO PSI efforts to promote open formats such as mzML and mzIdentML [46]. Vendor-specific formats, large file sizes, storage limitations, and inconsistent use of compression strategies further impede seamless integration. While PX repositories provide public access to proteomics data, the lack of mandatory complete submissions results in many partial datasets in proprietary or non-standardized formats [85].

Quantitative data representation also poses challenges. In DDA workflows, reuse is generally easier, as data can be reprocessed with freely available software. In contrast, DIA workflows present additional challenges, as quantification depends on spectral libraries and signal extraction algorithms. Making DIA data “FAIRable” requires formalized spectral library submission, adoption of open formats, and thorough documentation of input data and software versions. Similar challenges affect quantitative PTM datasets, where enrichment efficiency, site localization confidence, and modification-specific normalization influence measurements [30].

Legal and privacy considerations further constrain reuse. Even anonymized datasets may reveal unique protein signatures, raising re-identification risks [66,90,91,92,93]. Controlled-access models, careful metadata curation, and removal of identifiers can ensure GDPR compliance [94,95,96,97,98], but also create barriers to broad sharing and cross-platform integration.

## 7. Opportunities and Limitations of Machine Learning in Proteomics Data Reuse

The increasing volume and heterogeneity of proteomics datasets have made Machine Learning (ML) and AI indispensable tools across multiple steps of the analytical workflow. Their applications range from upstream spectra processing and peptide identification to downstream data analysis, multi-omics integration, and large scale data reuse [12,99].

At the level of peptide identification and spectral library generation, deep learning (DL) models can predict physicochemical properties of peptides directly from their amino acid sequences, including fragmentation spectra and retention times, enhancing peptide identification accuracy and enabling the generation of high-quality in silico spectral libraries for DIA experiments [100]. Community resources, such as ProteomicsML, support this process by providing curated, ML-ready datasets and standardized tutorials for peptide property prediction, facilitating systematic model evaluation across studies [99].

From data analysis, traditional statistical approaches like differential expression analysis remain widely used but often struggle with high-dimensional, heterogeneous datasets. Multivariate approaches, including principal component analysis (PCA), partial least square-discriminant analysis (PLS-DA), and its sparse variant (sPLS-DA), offer greater flexibility for classification and dimensionality reduction [101], enabling the identification of latent structure across multi-cohort datasets. Supervised ML classifiers, including random forests and support vector machines, extend this further by supporting feature selection tasks that identify protein subsets robustly associated with biological or clinical outcomes across independent studies. Embedded feature selection methods, including least absolute shrinkage and selection operator (LASSO) regularization and importance scores from tree-based models, reduce dimensionality while preserving the most informative features, improving model generalizability and interpretability in downstream analyses [101,102].

Beyond single-omics analysis, ML also facilitates the integration of proteomics with other omics layers. Graph-based ML approaches represent molecular entities and their interactions as networks, enabling the detection of functional relationships and pathway-level signals not accessible through conventional single-layer analyses, and supporting mechanistic insights relevant to tumor biology [103].

In the context of data reuse specifically, ML offers tools for imputation, normalization, feature selection, and cross-study integration [13,104] (Table 3).

Batch effects are systematic technical variations arising from differences in instruments, operators, sample preparation protocols, or acquisition settings. They represent one of the most significant sources of variability in proteomics data reuse, as they can obscure genuine biological differences and compromise cross-study comparability [105,106]. Dimensionality reduction methods such as PCA, uniform manifold approximation and projection (UMAP), and t-distributed stochastic neighbor embedding (t-SNE) are valuable for visualizing batch-related clustering and guiding quality assessment prior to integration, allowing the identification of datasets that are too heterogeneous to be directly combined [107]. ML models that incorporate batch information, such as neural network frameworks like BERNN, or mixed-effect models can learn batch-invariant representations and allow integration of heterogeneous datasets while maintaining biological variation [16,17]. However, batch correction alone does not resolve domain shifts arising from differences in patient populations, sample types, or experimental designs, which remain a major challenge for cross-study reuse. Moreover, overcorrection represents a concrete risk: when biological variation in interest is partially confounded with batch structure, aggressive correction may remove genuine biological signals alongside technical noise, leading to loss of clinically relevant information [107].

Missing values are an intrinsic feature of proteomics datasets and arise from several mechanisms, including stochastic sampling, detection limits, and study-specific technical factors [15]. From a data reuse perspective, missing values represent a critical obstacle: they reduce the overlap of quantified proteins across datasets, limiting the number of features available for cross-study integration and potentially introducing systematic bias if missingness is not random but correlated with sample type, acquisition strategy, or protein abundance range. Inappropriate imputation can introduce bias, especially when missingness patterns differ across studies. A thorough evaluation of intra-sample variability and missing values patterns is crucial to determine whether the absence of a protein reflects its true absence or indicates a signal below the limit of detection. ML-based approaches that exploit the global data structure, including self-supervised learning, can predict missing protein abundances while partially preserving biological variation [104]. Several ML imputation algorithms are available, broadly categorized into naïve imputation, feature-based imputation, global-based imputation, and ensemble methods [108,109]. The choice of imputation algorithm for imputation strategies should be carefully considered, taking into account the technology used, the experimental design, and the underlying missingness mechanisms, as careless imputation across heterogeneous datasets risks amplifying technical rather than biological signals. Furthermore, batch effects should be considered during imputation. Methods like HarmonizR combine structured imputation with batch effect correction, allowing for the harmonization of independent datasets for reuse [110]. A related but distinct challenge is the lack of comprehensive metadata annotation in public repositories. Systematic gaps in metadata provision, including missing information on labeling approaches, experimental design, and sample characteristics, substantially hinder reuse [111]. ML-based approaches can partially mitigate this issue by inferring missing metadata from raw data features, although this process remains error-prone and cannot substitute for rigorous annotation at the time of data deposition [112].

Heterogeneous experimental designs, including differences between label-free and isobaric labeling-based quantification, introduce additional structural incompatibilities that further complicate data reuse [12,14]. In these settings, direct combination of datasets is generally not advisable, as differences in quantitative structure, dynamic range, and proteome coverage can introduce substantial biases that obscure genuine biological signals. Cross-study normalization strategies can partially reduce systematic technical variation, but their effectiveness is limited when the underlying quantitative frameworks differ substantially across studies [12,110]. In such cases, domain adaptation approaches, a class of ML methods that learn shared representations across heterogeneous datasets, can support integration by identifying common patterns of protein variation that are consistent regardless of the quantification strategy used [113].

Small sample sizes represent an additional constraint in proteomics data reuse, particularly when integrating datasets from rare tumor subtypes or underrepresented patient populations. In this context, ML models trained on limited data tend to overfit, capturing dataset-specific patterns rather than generalizable biological signals. Transfer learning and multitask learning offer concrete strategies to mitigate this limitation: by pretraining on large, curated repositories like PRIDE Archive or CPTAC and subsequently fine-tuning them on smaller target datasets, models can transfer shared biological knowledge across studies, improving model generalizability and maximizing the analytical value of limited datasets [99].

The analytical power of ML, however, comes with important caveats. ML does not inherently ensure biological generalizability or clinical relevance and cannot compensate for insufficient experimental design: poor sample selection, lack of clinical annotation, or inadequate statistical power at the data generation stage cannot be corrected post hoc since poor-quality input data inevitably produce unreliable results (“garbage in, garbage out”). Models trained on heterogeneous or incompletely annotated datasets risk amplifying technical artifacts and capturing study, or research center-specific patterns, rather than disease-relevant biological signals. Data leakage between training and evaluation datasets can further lead to overoptimistic performance estimates. Without standardized preprocessing procedures, coherent clinical annotations, and validation strategies explicitly designed to assess cross-study generalization, ML-driven data reuse may generate technically sophisticated but biologically fragile models, limiting their reliability for patient stratification, biomarker discovery, and precision oncology.

Nevertheless, when these conditions are met, the translational potential of ML-enabled proteomics data reuse is increasingly evident. ML models can be trained on larger and more diverse cohorts than any single study could provide, improving generalizability across patient populations. A notable example is the integration of 183 datasets from PRIDE Archive with rigorous manual metadata curation, enabling a classifier to achieve high accuracy in tissue and cell type identification [12]. This work highlights the importance of data reuse and high-quality annotation in capturing biological signatures and developing tools with direct translational relevance, such as identifying tissue of origin in liquid biopsies and characterization of tissue-specific protein expression patterns relevant to tumor biology.

**Table 3:** Summary of Challenges and ML Strategies for Data Reuse.

Challenge	Impact on Data Reuse	ML Strategy
Missing values [15,110]	Bias in integrated datasets, overestimation of protein abundances	Structured imputation, self-supervised learning
Batch effects [105,106]	ML models learn technical variation instead of biology; overcorrection may remove genuine biological signals	Batch-aware models, embedding-based approaches, mixed-effect models, BERNN
Heterogeneous design [12,14]	Confounding biological signals, incompatible quantitative structures	Cross-study normalization, multitask learning
Low sample size [99]	Limited model generalizability, overfitting to dataset-specific patterns	Pretraining on public repositories, transfer learning, multitask learning
Technical variability [12,114]	Model overfitting to instrument artifacts	Instrument-aware modeling, domain adaptation
Low-quality data [13,104]	Amplification of noise and errors	Quality-aware preprocessing, uncertainty modeling

## 8. From Data Reuse to Biomarker Discovery and Therapeutic Targets

Single-cohort proteomics studies have generated numerous candidate biomarkers and therapeutic hypotheses in cancer research. However, many of these findings fail to generalize beyond the original study context, due to limited sample sizes, cohort-specific biases, and technical variability, as discussed in Chapter 6. In this context, systematic reuse of public proteomics datasets has emerged as a critical framework for validating, reassessing, and refining biomarker candidates, enabling extension beyond individual studies.

By leveraging datasets from large initiatives such as CPTAC or from archived tumor cohorts deposited in public repositories, researchers can identify protein-level alterations that are often more directly linked to cellular functions and therapeutic response than transcriptomic changes alone [5,7]. Importantly, proteomics enables the detection of distinct proteoforms, including isoform-specific and post-translationally modified protein species that are not resolved by genomic and transcriptomic approaches [77]. This distinction is particularly relevant in cancer, where different proteoforms of the same protein can exhibit distinct biological functions, subcellular localization and sensitivity to therapy [5].

In the context of data reuse, proteoform-aware resources and standards such as HPfA and ProForma 2.0 facilitate cross-cohort clinical analyses at proteoform resolution [63,89]. Integrating multiple independent datasets enables the identification of consistently regulated proteoforms that are more likely to reflect core disease mechanisms rather than cohort-specific artifacts. Reuse-driven studies in hepatocellular carcinoma and ovarian cancer, for example, have identified stable protein abundance patterns associated with clinical outcomes [5,7,115,116,117]. In this context, proteoform-aware analyses allow independent validation at a molecular resolution that extends beyond what single-cohort or transcriptomics studies can achieve [30,118].

### 8.1. Druggability and Membrane-Associated Therapeutic Targets

Protein druggability depends on multiple factors beyond abundance, including structural features, subcellular localization, and availability of biochemical properties [5]. Membrane-associated proteins are of particular interest because of their suitability for antibody-based therapies, including ADCs. The clinical success of human epidermal growth factor receptor 2 (HER2)-directed ADCs exemplifies how robust protein-level evidence can be translated into effective targeted therapies [119].

A cross-dataset perspective reduces the risk of prioritizing targets driven by cohort-specific effects and increases confidence in their clinical relevance [5]. Proteoform-level analyses enabled by data reuse further refine target selection by revealing isoform-specific differences in cellular localization or functional activity. Comparative analysis across multiple cohorts, combined with characterization of PTMs such as phosphorylation, glycosylation, and ubiquitination, highlights proteoforms that are consistently present and accessible at the cell surface [118,120,121].

Proteoform-aware reuse can also identify isoform-specific protein complexes and trafficking patterns that influence antibody binding, internalization, and payload delivery in a reproducible manner [120]. Similarly, cross-cohort comparison of ubiquitination patterns allows prioritization of proteoforms with sufficient membrane stability to serve as viable ADC targets [121]. By ensuring that therapeutic antibodies recognize disease-relevant proteoforms rather than canonical proteins, cross-dataset proteoform analysis supports more reliable target identification and early target triage, strengthening precision oncology strategies [118,121].

### 8.2. Proteomic Analysis of the Tumor Microenvironment

Tumor microenvironment plays an important role in shaping protein abundance patterns and modulating therapeutic response [122]. Proteomic analyses of tumor cores and invasive margins provide insights into microenvironment-associated signaling pathways and prognostic features. Recent advances in proteomics technologies have improved the detection of proteins involved in immune regulation, enabling more detailed characterization of tumor–immune interactions [123].

Reuse of proteomics datasets across multiple studies is particularly useful for tumor microenvironment research, as it enables the identification of immune and stromal signatures that are consistent across cohorts [52,124]. Incorporation of proteoform-level information further refines the interpretation of these data, since distinct isoforms and proteoforms may play specific roles in immune modulation, extracellular matrix organization, and cell–cell communication. In this context, proteoform-aware data reuse aligns with emerging concepts of proteoform medicine, in which distinct protein forms represent functionally and clinically relevant entities rather than interchangeable molecular surrogates [118].

## 9. Untangling Molecular Complexity and Data Issues in Colorectal and Prostate Cancer

Prostate cancer (PC) and Colorectal cancer (CRC) represent two significant examples of how molecular heterogeneity and data-related limitations influence the reuse and interpretation of proteomic datasets. Despite differences in tissue origin, disease progression and clinical management, both malignancies share recurring challenges related to experimental variability, incomplete metadata and limited reproducibility across studies. These factors constrain the effective integration of independent datasets and complicate the extraction of robust biological and clinical insights. The following sections discuss these aspects in the context of PC and CRC, highlighting both opportunities and current limitations of proteomics data reuse.

### 9.1. Data Reuse in Prostate Cancer

PC is the second most common malignancy in men worldwide, accounting for nearly 1.5 million new diagnoses and approximately 400,000 deaths in 2022, with marked differences in incidence across geographic regions and ethnic populations. The disease develops predominantly in older individuals, with a median age at diagnosis of 67 years, and has a strong inherited component [125].

Multiple studies have highlighted the potential of reusing publicly available proteomics datasets in PC, demonstrating that secondary analyses can generate biological insights beyond those reported in the original publications, while also advancing the development of harmonized computational workflows [126,127,128].

For instance, Jarnuczak and colleagues assembled the first meta-analysis of public cancer proteomics datasets, manually curating and reanalyzing over seven thousand MS runs from 11 large-scale studies deposited in PRIDE, MassIVE and the CPTAC portal, including PC primary tumor samples [127]. Using a harmonized MaxQuant-based pipeline with batch effect correction, the study identified lineage-specific proteomic signatures and confirmed the limited predictive value of mRNA levels for protein abundances. However, manual metadata curation remained the most time-consuming step, and restricted sample sizes per tumor type limited the depth of lineage-specific conclusions, underscoring that systematic proteomics data reuse is technically feasible but dependent on metadata quality.

In another study, Walzer and colleagues developed the first open, automated reanalysis pipeline for public SWATH-MS datasets [128]. While replicate reproducibility was comparable to the original studies, the overlap of differentially expressed proteins between original and reanalysed results was partial, with spectral library composition and protein inference identified as primary sources of discrepancy. The study demonstrated that robust cross-cohort integration requires both consistent spectral libraries and complete metadata annotation.

However, these studies also highlight that reproducibility and cross-cohort integration remain areas of active development.

In PC, general limitations of data reuse are further amplified by pronounced clinical heterogeneity. Differences in tumor stage, Prostate-Specific Antigen (PSA) levels, hormone sensitivity, and prior treatments directly influence protein abundance profiles and PTM patterns, complicating cross-cohort comparisons [128]. PSA remains the most widely used biomarker for PC detection and monitoring. However, its levels can also be elevated in benign conditions such as prostatitis or benign prostatic hyperplasia, limiting its ability to distinguish indolent from aggressive tumors and reduces its prognostic power [126,127,128]. Nevertheless, PSA constitutes a valuable clinical parameter and an important metadata feature when integrating proteomics data for patient stratification and interpretation [129,130]. Similarly, accurate reporting of Gleason score—a composite score between primary and secondary histological growth patterns reflecting tumor aggressiveness—is essential when integrating proteomics measurements with clinical characteristics [131,132,133].

On the technical side, heterogeneity in quantification strategies constitutes a challenge. This issue is exemplified by TMT- based dataset PXD013422 in PRIDE Archive, in which the samples from each experimental group were pooled and labeled with a single reporter [134]. The measured protein abundances represent aggregate values for each group, and they do not reflect individual patient variability. Another example comes from SILAC- and Super-SILAC-based datasets in which the output analysis is constituted by protein ratios relative to a reference standard rather than abundance values—as for the dataset PXD003430 [135]. From a data reuse perspective, the first approach limits the direct comparison with datasets in which single samples are labeled and quantified separately, while the second type of dataset cannot be directly compared with studies in which absolute abundances are reported. These experimental design characteristics must therefore be carefully considered when performing cross-study integration.

Regarding the metadata-related issues, in some studies clinical variables such as PSA have not been collected during cohort recruitment because they are not required for the primary experimental objectives, like PXD010744 and PXD003430 [135,136]. Although this does not necessarily affect the conclusions of the original study, the absence of these measurements becomes a limitation in the context of data reuse. This complicates cross-study comparisons and limits the interpretation of protein abundance patterns in relation to clinically relevant indicators of disease progression.

Among the initiatives that have most substantially expanded the landscape of reusable proteomic resources in oncology, the Pan-Cancer Proteome Atlas (TPCPA) reported by Knol and colleagues is significant [84]. Through a DIA-MS workflow applied uniformly across 999 primary tumors from 22 cancer types, the study produced a reference compendium of nearly 10,000 proteins, deposited in PRIDE Archive (PXD054790) and made interactively accessible through a dedicated data portal.

At the disease-specific level, Sun and colleagues established one of the most comprehensive proteomic resources for primary PC to date by quantifying more than 10,000 proteins across 306 individually profiled FFPE specimens using DIA-MS [137]. Beyond the scale of protein coverage, the study defined three molecularly distinct PC subtypes with significant differences in clinical outcome, mapped proteomic alterations associated with ISUP grade groups, and identified NUDT5 and SEPTIN8 as candidate therapeutic targets. A 16-protein prognostic classifier for biochemical recurrence was developed and validated across six independent published cohorts and one additional biopsy-level dataset quantified by targeted proteomics, demonstrating both the biological depth achievable in clinical PC proteomics and the cross-study compatibility of well-annotated, individual-level proteomic data.

Overall, the PC proteomics landscape has progressed from single-cohort discovery studies to large-scale, individually annotated resources amenable to cross-study integration, with early reuse efforts demonstrating both the biological value and the technical feasibility of secondary analyses. Nevertheless, quantification heterogeneity, inconsistent metadata annotation, and limited clinical variable reporting remain the principal barriers to systematic data reuse in this tumor type.

### 9.2. Data Reuse in Colorectal Cancer

CRC is the third most common cancer worldwide, representing about 10% of all cancer cases. Although advances in molecular profiling have improved disease characterization, important gaps remain in understanding tumorigenesis and the regulatory mechanisms underlying disease progression [138,139].

Large-scale proteomic studies in solid tumors proteomics have played a pivotal role in generating datasets that later became valuable resources for systematic data reuse: a notable milestone in this transition is represented by the Pan-Cancer Proteome Atlas described by Knol and colleagues [84], which systematically profiled thousands of proteins across multiple tumor types (including CRC) using standardized DIA-MS workflows. By depositing the resulting datasets in public repositories and providing interactive exploration platforms, this initiative transformed raw proteomic measurements into structured, queryable resources that can be reanalyzed and integrated across studies.

A great contribution recently published illustrates the feasibility of large-scale protein profiling in CRC by establishing well-characterized cohorts that subsequently became an attractive starting point for secondary analyses: Feliu and colleagues [140] showed that high-quality proteomic data can be reliably generated from Formalin-Fixed Paraffin-Embedded samples and used to refine consensus molecular subtypes through probabilistic graphical modeling. Importantly, these approaches revealed proteomic heterogeneity within established transcriptional subtypes, highlighting biological features that are not captured by transcriptomics alone and expanding the spectrum of clinically actionable targets. These outcomes underscore how proteomic data reuse can uncover clinically relevant targets, guiding the development of next-generation immunotherapies for CRC.

Several studies illustrate the potential of proteomics data reuse in CRC. Robles and colleagues conducted an integrated meta-analysis by reanalyzing twelve publicly available proteomics CRC datasets [7]. By using data from both solid and liquid biopsies, researchers were able to validate an existing gene signature and identify five novel prognostic biomarkers detectable in blood. Among these, CD14 and TXNDC5 emerged as key proteins with high clinical value for predicting disease progression. This outcome demonstrates how data reuse not only strengthens biomarker validation but also enables the discovery of clinically relevant targets, further enhancing patient stratification and precision oncology strategies. Interestingly, a recent study highlights the potential of CDH17 as an ideal surface protein target for CRC immunotherapy [7]. Through an integrated analysis of transcriptomic and proteomic data, the research identified CDH17 as a highly expressed protein in CRC cells, yet absent in critical normal tissues such as the lungs and brain, reducing the risk of severe toxicity in immunotherapy. Unlike other well-established tumor markers (e.g., carcinoembryonic antigen), CDH17 is not expressed in the lungs, making it a safer alternative for antibody-based treatments [141]. Interestingly, CDH17 is currently being explored as a therapeutic target in gastrointestinal malignancies. In particular, the anti-CDH17 ADC Cabotamig (ARB202) is currently under evaluation in a phase I clinical trial in patients with advanced gastrointestinal cancers (NCT05411133) [142].

Still, the overall number of reuse-driven CRC studies is influenced by constraints related to how samples are defined and how cohorts are structured in single studies, underscoring an opportunity for broader validation and extension of candidate biomarkers across independent cohorts [7,141,143,144]. In many public repositories, tumor samples are imprecisely annotated with respect, for example, to the anatomical origin, and are not consistently paired with the corresponding normal adjacent tissues. This lack of contextual information complicates the interpretation of proteomic profiles, as tumors from different anatomical regions of the colon and rectum may exhibit distinct molecular characteristics. For example, the publicly available CRC proteomics datasets from PRIDE Archive PXD009475 as well as PXD001676 include proteomic profiles from a very few matched tumor/normal tissue pairs, a cohort size that, while sufficient for the original discovery purpose, can limit cross-study integration [145,146]. In fact, studies with limited patient numbers increase statistical uncertainty and amplifies the inter-patient heterogeneity. Indeed, integrating multiple datasets can mitigate these limitations by increasing the effective cohort size; however, when individual studies include only a few patients, cross-study integration becomes more sensitive to inter-patient variability and methodological differences, such as inclusion criteria, sample processing, and data acquisition protocols. As a result, small cohorts can contribute to integrative analyses only when sufficient metadata and methodological details are available, allowing datasets to be reliably harmonized and combined with compatible studies. Unfortunately, the variability in sample composition, missing molecular subtypes annotations in different Tumor-Node-Metastasis stage and/or enrichment techniques among datasets complicate direct comparisons with other datasets and can distort proteomic results. For instance, in the PXD019504 cohort, P4HA1 was identified as a prognostic biomarker, but only in microsatellite stability tumors; the lack of MMR annotation in other datasets would have diluted or misattributed this finding in cross-study integrations [147].

Overall, careful selection of compatible datasets and proper harmonization of metadata are essential to mitigate these limitations. When done systematically, such approaches can yield meaningful and robust biological insights.

### 9.3. Critical Considerations for Proteomics Data Reuse in Solid Tumor Research

From a practical perspective, the integration of proteomics datasets in colorectal and prostate cancer depends largely on the methodological compatibility between studies. Two complementary strategies can therefore be considered: integration at the level of protein quantification when studies are methodologically comparable, and integration at the level of biological interpretation when datasets originate from heterogeneous experimental designs.

Integration is most straightforward when proteomics datasets share comparable experimental designs. Label-free quantification studies or DIA-based acquisition workflows can be reprocessed using consistent database search parameters, filtering criteria, and normalization procedures, enabling the generation of comparable protein abundance matrices across cohorts. When structured clinical annotations are available, harmonized datasets can also support clinically informed patient stratification, thus enabling the integration of larger cohorts and improving the statistical power required to identify biologically and clinically relevant protein signatures.

Integration becomes considerably more challenging when datasets derive from different experimental strategies. DDA-based label-free experiments, TMT multiplex quantification, and enrichment-driven workflows differ in quantitative structure, proteome coverage, and dynamic range, which can introduce substantial biases if datasets are directly combined. In such cases, we suggest adopting a layered integration strategy in which datasets are first harmonized within methodologically comparable groups or stratified according to shared biological or clinical features, and then compared across studies. This approach shifts the integration toward the level of biological interpretation, focusing on concordant protein alterations, pathway-level signals, or functional modules consistently detected across independent datasets.

## 10. Conclusions and Future Perspectives

Proteomics data reuse in solid tumors holds clear promise, yet its effective application remains constrained by a combination of biological, technical and infrastructural constraints. Despite significant advances in proteomic technologies and the rapid expansion of public repositories, increased data availability has not yet translated into reliable or widespread reuse.

A central limitation arises from the mismatch between the biological complexity of solid tumors and the way proteomics data are commonly generated and summarized. Solid tumors are shaped by spatial heterogeneity, microenvironmental influences, and dynamic regulation at the proteoform level. Still, many reuse efforts rely on protein-centric summaries that mask this complexity. PTMs represent a clear example: while phosphorylation-dependent signaling has been extensively investigated, other PTM classes remain sparsely annotated, inconsistently quantified, or entirely absent from large-scale datasets. This imbalance limits the range of biological questions that can be addressed through reuse and introduces biases in downstream interpretation.

Technical and infrastructural factors further limit effective reuse. Differences in acquisition strategies, enrichment protocols, and quantification approaches introduce variability that cannot be resolved retrospectively without comprehensive and standardized metadata. Even when raw data are accessible, incomplete annotation and heterogeneous data structures often prevent meaningful cross-cohort integration. Under these conditions, reuse typically requires layered integration strategies that combine methodological harmonization with higher-level biological comparison. Without such strategies, data reuse may amplify technical artifacts rather than reveal robust biological signals.

These limitations are particularly critical in translational contexts. For instance, target selection for ADC development depends not only on protein abundance, but also on proteoform composition, PTM patterns, and spatial accessibility at the tumor cell surface. Overgeneralization of proteomics findings across tumor types or cohorts may obscure patient-specific molecular features that are crucial for developing individualized therapeutic strategies. Precision oncology therefore requires reuse strategies that preserve, rather than flatten, molecular heterogeneity.

ML approaches offer new opportunities to integrate heterogeneous proteomics datasets by addressing inconsistencies and discrepancies across studies. However, their effectiveness depends on the quality and structure of the underlying data. Without careful model design and biological interpretation, these methods may amplify technical variation rather than resolve it. The reanalysis of CRC and PC datasets highlights these challenges, showing how variation in data depth, experimental design and annotation quality directly limits reproducibility and translational relevance.

Future progress will therefore require a conceptual shift in perspective. Data reuse should be considered an integral component of experimental design, not merely a secondary opportunity. While improvements in data standards, metadata consistency, and FAIR-compliant repositories are important, they are not sufficient on their own.

Effective reuse strategies hold substantial potential, but their success critically depends on a deep understanding of the biological context, the specific research question, and the molecular resolution required. To achieve reliable and meaningful results, researchers must possess both advanced technical skills and strong biological expertise. These skills are essential not only to interpret complex datasets but, importantly, to develop a critical perspective towards publicly available material, identifying limitations, recognizing poorly designed or low-quality datasets, and avoiding analyses that could mislead results.

In conclusion, the value of proteomics data reuse in solid tumor research does not simply lie in the growing number of available datasets, but in the ability to interrogate them with biological precision. Strengthening reuse will require both technical innovations and a cultural shift towards more open, standardized, collaborative research practices, coupled with biologically informed data generation. Addressing these challenges will enhance the robustness and impact of proteomic research and contribute to the development of more personalized and effective cancer therapies.

## Figures and Tables

**Table 1:** Major proteomics technologies, analytical strengths, and sources of technical variability.

	Method/Strategy	Main Strengths	Key Limitations/Sources of Variability
**MS-based** **Proteomics** [27,28,29,30,31,32,33]	Top–down proteomics	Direct analysis of intact proteins; characterization of proteoforms and PTMs	Technically demanding; reduced sensitivity for large proteins; limited proteome coverage
	2DE–LC–MS/MS	High-resolution separation of proteoforms prior to identification	Gel-to-gel variability; limited throughput; labor-intensive workflows
	Bottom–up proteomics	High-sensitivity and proteome-wide coverage; widely used for discovery studies	Loss of proteoform-level information and ambiguous protein inference
**Data acquisition strategies** [27,28,29,30]	DDA	Efficient discovery workflows	Stochastic precursor selection leading to missing values and limited reproducibility across runs
	DIA	Improved reproducibility and quantitative consistency across large sample cohorts	Computational complexity and dependence on spectral libraries
	SWATH-MS	Comprehensive fragment ion maps; retrospective and PTM-focused analyses	Limited standardization of acquisition parameters across laboratories
	Targeted MS (SRM/MRM, PRM)	Highly reproducible and precise quantification	Restricted to predefined protein targets; limited discovery applications
**Quantification Strategies** [29,34,35,36,37]	Label-based approaches (ICAT, iTRAQ, TMT, SILAC)	High quantitative precision and multiplexing capability	Ratio compression; incomplete labeling; limited cross-study comparability
	LFQ	Flexible and scalable for large datasets	Sensitive to experimental variability; missing values; chromatographic fluctuations
	AQUA, PSAQ, QconCAT	Accurate absolute protein quantification	High cost; extensive assay optimization; predefined targets required
**Affinity-based proteomics** [30,38,39,40,41,42,43,44,45]	Antibody microarrays/RPPA	Targeted protein detection with moderate throughput	Antibody cross-reactivity and dependence on antibody quality
	PEA (Olink)	High sensitivity and multiplexing for low-abundance proteins	Restricted to predefined protein panels
	Bead-based assays (Luminex, Simoa, SOMAscan)	High multiplexing and sensitivity	Matrix effects; analyte-specific variability; limited cross-platform harmonization

**Table 2:** Core ProteomeXchange partner proteomics repositories and their characteristics.

Repository	Main Focus	Data Types	Total Datasets ^a^	Metadata and Standardization	Reuse Support	Strengths for Data Reuse
PRIDE [11,67,68,71,75,76]	General MS-based proteomics	DDA, DIA, top–down, peptidomics, crosslinking	>55,000	SDRF–Proteomics, experimental design, PX-compliant	Quantms, OpenMS, DIA-NN, USI, PSM access	Central hub for reuse, broad data coverage, integration with UniProt and Expression Atlas
MassIVE [68,78]	Quantitative and community-scale MS	DDA, DIA, targeted and untargeted	>18,000	Experimental design, scripts, intermediate files	MassIVE.quant, MSstats, MassIVE-KB	Enables alternative reanalyses, strong provenance, reusable spectral libraries
jPOSTrepo jPOSTdb [77,79,80]	General proteomics with standardized reanalysis	DDA, DIA, SRM, PRM, 2DE, antibody-based	>3800	SDRF metadata, KEGG annotations	UniScore-based reanalysis; jPOSTdb integration	Consistent reanalysis; curated protein-level results
iProX [77,82]	National and international proteomics	Mainly DDA, raw and processed MS data	>6700	SDRF-Proteomics, PX-compliant XML metadata	DDA reanalysis pipeline	Strong metadata completeness, interoperability with PX
Panorama Public [77,81]	Targeted quantitative proteomics	Skyline-based targeted MS	>650	Skyline document structure	Native Skyline integration	Detailed quantitative reuse, chromatogram-level inspection
PeptideAtlas [9]	Reanalysis and aggregation of public MS data	Reprocessed DDA MS data	Community-scale builds	Standardized pipelines; strict FDR	Systematic reanalysis of PX data	Cross-study comparability; protein existence evidence

^a^ Dataset counts refer to repository statistics available as of January 2026.

## Data Availability

Data are contained within the article.

## Abbreviations

The following abbreviations are used in this manuscript:

2DE	Two-Dimensional gel Electrophoresis
ADC	Antibody–Drug Conjugate
AI	Artificial Intelligence
AQUA	Absolute QUAntification
CD14	Cluster of Differentiation 14
CDH17	Cadherin 17
CZE-MS/MS	Capillary Zone Electrophoresis coupled with Tandem Mass Spectrometry
CRC	Colorectal Cancer
CPTAC	Clinical Proteomic Tumor Analysis Consortium
CTDP	Consortium for Top-Down Proteomics
DDA	Data-Dependent Acquisition
DIA	Data-Independent Acquisition
FAIR	Findable, Accessible, Interoperable, Reusable
FDR	False Discovery Rate
FAIMS	Field Asymmetric Ion Mobility Spectrometry
GELFrEE-MS	Gel-Eluted Liquid Fraction Entrapment Electrophoresis Mass Spectrometry
GDPR	General Data Protection Regulation
HDF5	Hierarchical Data Format version 5
HER2	Human Epidermal growth factor Receptor 2
HPA	Human Protein Atlas
HPfA	Human Proteoform Atlas
HPP	Human Proteoform Project
HUPO PSI	Human Proteome Organization Proteomics Standards Initiative
ICAT	Isotope-Coded Affinity Tag
IDF	Identification Definition Format
iTRAQ	Isobaric Tags for Relative and Absolute Quantification
KEGG	Kyoto Encyclopedia of Genes and Genomes
LC-MS/MS	Liquid Chromatography coupled with Tandem Mass Spectrometry
LFQ	Label-Free Quantification
MAGE-TAB	MicroArray Gene Expression Tabular
MassIVE	Mass Spectrometry Interactive Virtual Environment
MassIVE-KB	MassIVE Knowledge Base
ML	Machine Learning
MMR	Mismatch Repair
MRC2	Mannose Receptor C-Type 2
MRM	Multiple Reaction Monitoring
MS	Mass Spectrometry
PEA	Proximity Extension Assay
PC	Prostate Cancer
PCA	Principal Component Analysis
PLS-DA	Partial Least Squares Discriminant Analysis
PPIA	Peeptidyl-prolyl cis-trans Isomerase A
PRDX1	Peroxiredoxin-1
PRIDE	PRoteomics IDEntifications Archive
PRM	Parallel Reaction Monitoring
PSA	Prostate Specific Antigen
PSAQ	Protein Standard Absolute Quantification
PX	ProteomeXchange
PTM	Post-Translational Modification
QconCAT	Quantification conCATemer
RPPA	Reverse-Phase Protein Array
SAAVs	Single Amino Acid Variants
SDRF	Sample and Data Relationship Format
SILAC	Stable Isotope Labeling by Amino acids in Cell culture
sPLS-DA	Sparse Partial Least Squares Discriminant Analysis
SRM	Selected Reaction Monitoring
SWATH-MS	Sequential Window Acquisition of all Theoretical Mass Spectra
T-SNE	t-Distributed Stochastic Neighbor Embedding
TCGA	The Cancer Genome Atlas Program
TMT	Tandem Mass Tag
TPCPA	Pan-Cancer Proteome Atlas
TXNDC5	Thioredoxin Domain-Containing 5
UMAP	Uniform Manifold Approximation and Projection
USI	Universal Spectrum Identifier
